# Anti-tumor target screening of sea cucumber saponin Frondoside A: a bioinformatics and molecular docking analysis

**DOI:** 10.3389/fonc.2023.1307838

**Published:** 2023-12-08

**Authors:** Guangchun Liu, Shenglin Zhang, Ruoyan Lin, Xudong Cao, Lihong Yuan

**Affiliations:** ^1^ School of Biosciences and Biopharmaceutics, Guangdong Pharmaceutical University, Guangzhou, China; ^2^ Deparment of Chemical and Biological Engineering, University of Ottawa, Ottawa, ON, Canada

**Keywords:** Frondoside A, antitumor, integrated bioinformatical analysis, molecular docking, therapeutic target genes

## Abstract

Cancer remains the leading cause of death worldwide. In spite of significant advances in targeted and immunotherapeutic approaches, clinical outcomes for cancer remain poor. The aim of the present study was to investigate the potential mechanisms and therapeutic targets of Frondoside A for the treatment of liver, pancreatic, and bladder cancers. The data presented in our study demonstrated that Frondoside A reduced the viability and migration of HepG2, Panc02, and UM-UC-3 cancer cell *in vitro*. Moreover, we utilized the GEO database to screen and identify for differentially expressed genes (DEGs) in liver, pancreatic, and bladder cancers, which resulted in the identification of 714, 357, and 101 DEGs, respectively. Gene Ontology (GO) analysis and Kyoto Encyclopedia of Genes and Genomes (KEGG) pathway annotation were performed using the Metascape database for DEGs that were significantly associated with cancer development. The protein-protein interaction (PPI) networks of the identified DEGs in liver, pancreatic, and bladder cancers were analyzed using Cytoscape 3.9.0 software, and subsequently identified potential key genes that were associated with these networks. Subsequently, their prognostic values were assessed by gene expression level analysis and Kaplan-Meier survival analysis (GEPIA). Furthermore, we utilized TIMER 2.0 to investigate the correlation between the expression of the identified key gene and cancer immune infiltration. Finally, molecular docking simulations were performed to assess the affinity of Frondoside A and key genes. Our results showed a significant correlation between these DEGs and cancer progression. Combined, these analyses revealed that Frondoside A involves in the regulation of multiple pathways, such as drug metabolism, cell cycle in liver cancer by inhibiting the expression of CDK1, TOP2A, CDC20, and KIF20A, and regulates protein digestion and absorption, receptor interaction in pancreatic cancer by down-regulation of ASPM, TOP2A, DLGAP5, TPX2, KIF23, MELK, LAMA3, and ANLN. While in bladder cancer, Frondoside A regulates muscle contraction, complement and coagulation cascade by increase FLNC expression. In conclusion, the present study offers valuable insights into the molecular mechanism underlying the anticancer effects of Frondoside A, and suggests that Frondoside A can be used as a functional food supplement or further developed as a natural anti-cancer drug.

## 1 Introduction

Cancer remains the predominant cause of mortality globally. In 2020, according to the GLOBOCAN report, the annual incidence of new cancer cases is estimated to be 19.3 million, with nearly 10.0 million cancer-related deaths (1). The projected incidence of new cancer cases in China and the USA for the year 2022 is around 4,820,000 and 2,370,000, respectively, with estimated cancer-related deaths of approximately 3,210,000 and 640,000 in these two countries (2). In the past decades, many basic and clinical studies have investigated underlying mechanisms of cancer formation and progression leading to advances in the molecular biology and diagnosis of cancers. However, despite these advancements, the incidence and mortality rates of cancer continue to exhibit high levels, and the available treatment options remain limited in their efficacy and scope (3). While there are many treatment options currently available for cancer patients – and many are clinically effective – these treatment options are known to have significant side effects (4, 5). Consequently, the development of novel and efficacious therapeutic agents for the treatment of cancer constitutes an urgent requirement. In general, natural products have served as a principal reservoir of compounds for the management of a diverse spectrum of cancers, presenting encouraging prospects for examining not only novel anticancer agents but also unexplored and potentially significant mechanisms of action (6). Marine organisms have emerged as a recent research focus for the identification of novel compounds that hold potential in the prevention and treatment of cancer (7), and there is growing interest in exploiting the diverse and complex marine organisms for rational drug discovery (8). The advancement of technology and extensive research on marine natural products have culminated in the identification of a novel cohort of anti-cancer drugs, and a number of marine biological extracts are currently being studied in clinical trials after showing anti-cancer activity in various preclinical studies (7, 9, 10). So far, 20 drugs from marine sources are already in clinical use (10).

**Figure 1:**
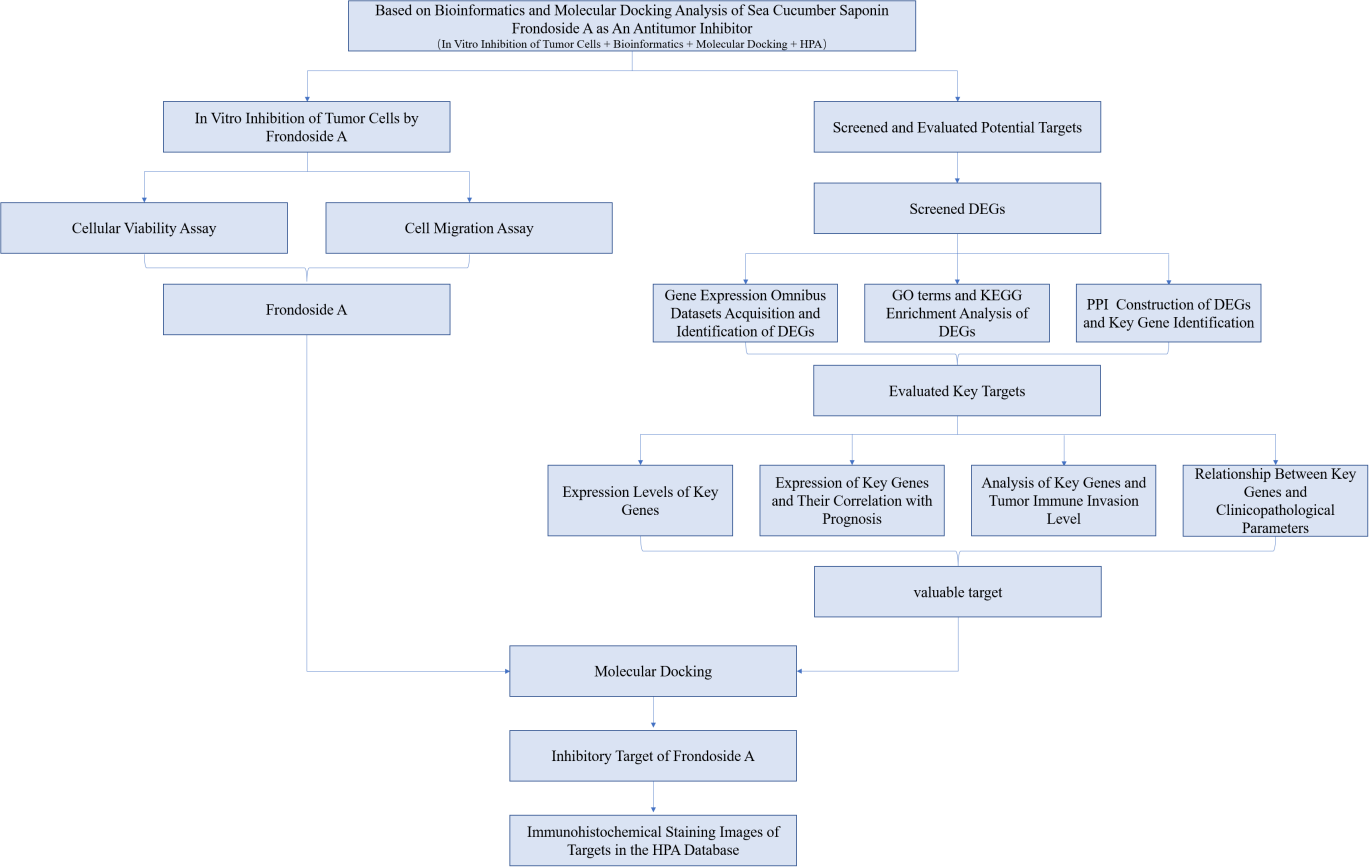
The flow chart for whole process analysis of articles. Preliminary analysis of the anticancer activity of Frondoside A by *in vitro* cell activity assay and screening of potential anticancer targets by combining bioinformatics and molecular docking methods.

To date, the exploration for anticancer agents in diverse marine phyla has unveiled numerous active compounds (11). The echinoderms, which exclusively inhabit the marine milieu and encompass sea stars, sea urchins, sand dollars, sea cucumbers, and sea lilies, have garnered significant attention as a subject of interest, amongst which sea cucumbers are of particularly interesting as they have been used as dietary remedies for cancer, inflammation, and other diseases for centuries (12). For example, triterpenoid glycosides from diverse species of sea cucumbers have been recognized for their potential anti-cancer properties (13). Frondoside A, a specific triterpenoid saponin, has recently gained significant attention due to its demonstrated potent anti-cancer effects against various types of solid malignancies and leukemias (14). However, few studies have attempted either Frondoside A as a potential treatment option for liver, pancreatic, and bladder cancers or investigated their mechanisms of action. In this study, we analyzed the *in vitro* antitumor activity of Frondoside A and its potential targets, and mechanisms of action related to the treatment of liver, pancreatic and bladder cancers using *in vitro* cellular assays, bioinformatics and molecular docking techniques. The full analytical work flow of this study is shown in **Figure 1**.

## 2 Materials and methods

### 2.1 Reagents

Triterpene glycoside Frondosid A (extracted from Cucumaria frondosa, purity > 98% determined by HPLC) was purchased from Kerafast (Boston, MA). Cell Counting Kit-8 (CCK-8) was obtained from US EVERBRIGHT^®^INC., Suzhou, Jiangsu, China. DMEM medium, MEM medium, fetal bovine serum (FBS), and phosphate buffer solution (PBS, pH 7.4) were purchased from Thermo Fisher Scientific (Waltham, MA. Finally, penicillin (100 Units/mL), streptomycin (100 µg/mL), 0.25% Trypsin (×1), and Nonessential Amino Acid Solution (NAAS, 100X) were obtained fromGibco (Grand Island, NY) were used in the study Chemotherapeutic agent Epirubicin hydrochloride (EPI), a commonly used anti-tumor drug to treat solid tumors, for instance liver cancer, breast cancer, and bladder cancer, was purchased from Aladdin.

### 2.2 Cell lines and cell cultures

Three cancer cell lines were used in this study, including Human liver cancer cell HepG2 (ATCC^®^ No. HB-8065), Human pancreatic cancer cell Panc02 (ATCC^®^ No. CRL-2553) and Human bladder cancer cell UM-UC-3 (ATCC^®^ No. CRL-1749). Both HepG2 and Panc02 cells were incubated with DMEM medium supplemented with 10% FBS, and UM-UC-3 cells were cultured in MEM medium supplemented 1% NAAS. Moreover, three cell lines were supplemented with 10% FBS, 100 Units/mL of penicillin, and 100 μg/mL, and incubated at 37°C with 5% CO_2_ atmosphere and 100% humidity.

### 2.3 Cell viability assay

To evaluate the cytotoxicity of drugs of interest, HepG2, Panc02, and UM-UC-3 cells were seeded in 96-well plates at a density of 5000 cells per well and incubated overnight. Triplicate treatments with increasing concentrations of Frondoside A were administered to the cells for 48 hours, while EPI (10 μM) and PBS (pH 7.4) were used as positive and blank controls, respectively. Subsequently, PBS (pH 7.4) was used to carefully wash the cells, after which 10% CCK-8 reagent was added, and the cultures were incubated for 3 hours. The plates were analyzed at 450 nm utilizing a microplate reader (Bio-Rad, Hercules, CA). Cell viabilities were expressed as proportional viabilities (%) normalized against the blank control and assumed 100% survival. The half maximal inhibitory concentration (IC_50_) values of Frondoside A for HepG2, Panc02, and UM-UC-3 cell lines were also determined.

### 2.4 Cell migration assay

Confluent cell monolayers of HepG2, Panc02, and UM-UC-3 cells were cultivated in 6-well tissue culture plates. A plastic micropipette tip was used to carefully scratch the confluent cell monolayers, and the cell cultures were subsequently washed twice with PBS (pH 7.4). The cells were cultured in their respective cell culture media that contained 1% FBS and supplemented with Frondoside A at their respective IC_50_ concentrations for 24 h. The widths of the wound in the cultures were documented using an Olympus inverted microscope at 0, 12, and 24 h after the wound. Finally, the scratched area was measured using a NIH Image J (Bethesda, MD), and the degree of wound closure was calculated.

### 2.5 Microarray data collection

The Gene Expression Omnibus (GEO) database (https://www.ncbi.nlm.nih.gov/geo/) is a publicly available functional genomics resource that provides high-throughput gene expression data, chips, and microarrays (15). For the relevant tumors, gene expression profile data was acquired from the GEO database. The inclusion criteria were: (1) The specimens were obtained from patients with human liver cancer, pancreatic cancer, and bladder cancer; (2) The study population was comprised of both tumor patients and normal controls; (3) The study design was “expression profiling by array”. Finally, we downloaded liver cancer-related datasets (GSE14520 and GSE60502), pancreatic cancer datasets (GSE16515 and GSE28735), and bladder cancer datasets (GSE13507, GSE23732, and GSE37815) from the GEO database.

### 2.6 DEGs identification and data visualization

The GEO2R (https://www.ncbi.nlm.nih.gov/geo/geo2r/) is an online analytical tool that is utilized for the identification of differently expressed genes (DEGs) between tumor and healthy controls. The data was categorized into tumor and normal groups and analyzed based on |log_2_FC| ≥ 1.0 and adj. *p* value < 0.05, respectively. Following the screening of the DEGs within each dataset, the Venny2.0 online tool (https://bioinfogp.cnb.csic.es/tools/venny/) was employed to identify common targets within the relevant datasets, and the overlapping DEGs were retained for further analysis.

### 2.7 Gene ontology terms and KEGG enrichment analysis of DEGs

In order to annotate the DEGs identified from the aforementioned comparison groups in a functional manner, GO term annotation and KEGG pathway analysis were conducted with the use of Metascape. Metascape (https://metascape.org/gp/index.html#/main/step1) (16) is a comprehensive analytical website that incorporates functional enrichment, gene annotation, interactome analysis, and membership search, leveraging more than 40 independent knowledge bases. KEGG is a database resource used for elucidating high-level features and effects of biological systems, while GO is a widely used bioinformatics program for high-quality functional gene annotation based on biological processes (BP), molecular functions (MF), and cellular components (CC). Metascape was utilized with a minimum overlap of 3 and a minimum richness of 1.5 as screening criteria to determine the characteristics of DEGs. A significance level of *p* < 0.01 was considered statistically significant, and GraphPad Prism 9.0 software was used to generate bar graphs to visually represent the obtained results.

### 2.8 Protein-protein interaction network construction and key gene identification

The intersection targets that were obtained were subjected to analysis and construction of Protein-Protein Interaction (PPI) networks using the STRING database (https://string-db.org/). The STRING database is one of the most informative databases for studying protein interactions at this stage, which evaluates and integrates information on known and predicted protein interactions to form a comprehensive protein network covering > 1,100 organisms (17). The STRING database utilizes probability calculations of various evidence channels, including the correction of random interaction probability, to construct PPI networks. The resulting composite score is assigned based on the level of confidence, with a score of 0.4 indicating medium confidence, 0.7 indicating high confidence, and 0.9 indicating the highest level of confidence. In this study, a PPI network with a score of 0.9, i.e., the highest confidence network, was used to import potential targets into the STRING database and select “Homo Sapiens” species to obtain target protein interaction relationships.

### 2.9 Expression analysis of key genes

GEPIA2.0 (http://gepia2.cancer-pku.cn/#index) is a newly developed web server for cancer and normal gene expression analysis and the interactive analysis with rich analysis functions, such as tumor/normal differential expression analysis, survival analysis, gene correlation analysis, and downscaling analysis (18). This study will use the GEPIA2.0 database to analyze the obtained key genes and initially validate their expression differences between tumor and normal tissues.

### 2.10 Prognostic analysis of survival of key genes

In this study, GEPIA2.0 was utilized to examine the correlation between the expression of key genes in tumors and overall survival (OS) (18). The statistical analysis was performed by calculating risk ratios (HR) and *p* values with 95% interval confidence (IC) using a Log-rank test. Finally, genes with insignificant differences were excluded for further analysis to screen for key genes with a significant expression.

### 2.11 Analysis of key genes and tumor immune invasion level

The Tumor Immunity Estimation Resource (TIMER) database contains 10,897 samples from The Cancer Genome Atlas (TCGA) for 32 cancer types (19). In this study, the correlation between key genes and the degree of immune cell infiltration, including B cells, CD8^+^ T cells, CD4^+^ T cells, neutrophils, macrophages, and dendritic cells, was investigated using the TIMER2 database (http://timer.cistrome.org/). The tumor purity of PCs was adjusted prior to analysis. Key genes were input into the “gene module” to generate scatter plots, which were used to observe the relationship between gene expression and the level of tumor immune invasion.

### 2.12 Molecular docking

To confirm the binding of Frondoside A to the predicted key targets, the structural formula of Frondoside A was retrieved and downloaded from the PubChem database (https://www.ncbi.nlm.nih.gov/pccompound) in mol format. The structure was optimized using Chem3D 14.0 software with MM2 force field for energy minimization and saved in pdb format. Three-dimensional structures of CDK1 (PDB ID: 4YC6) (20), ASPM (PDB ID: 3QBT) (21), BUB1 (PDB ID: 4QPM) (22), KIF20A (PDB ID: 5LEF) (23), TOP2A (PDB ID:5NNE) (24), TPX2 (PDB ID:4C3P) (25), KIF23 (PDB ID: 3VHX) (26), MELK (PDB ID:5TWL) (27), and FLNC (PDB ID: 7OUU) were obtained from RCSB PDB database (https://www.rcsb.org/) (28). The proteins were preprocessed with PyMOL software for dehydration, hydrogenation, and removal of irrelevant ligands. The Grid Box parameters were set with the receptor as the center, and molecular docking analysis was performed with AutoDock Vina 1.1.2. The conformation with the lowest free energy was selected and visualized with PyMOL (29). A binding free energy of < -5 kcal/mol indicates that the target has some binding activity to the compound (30), and the lower the binding energy, the more stable the receptor-ligand binding (31).

### 2.13 Statistical analysis

The experiments were conducted in triplicate, and the data were presented as mean ± standard deviation (SD). Statistical analysis was performed using unpaired Student’s t-test and one-way analysis of variance (ANOVA). Results with a *p*-value of less than 0.05 were considered statistically significant.

## 3 Results

### 3.1 *In vitro* inhibition of tumor cells by Frondoside A

**Figure 2:**
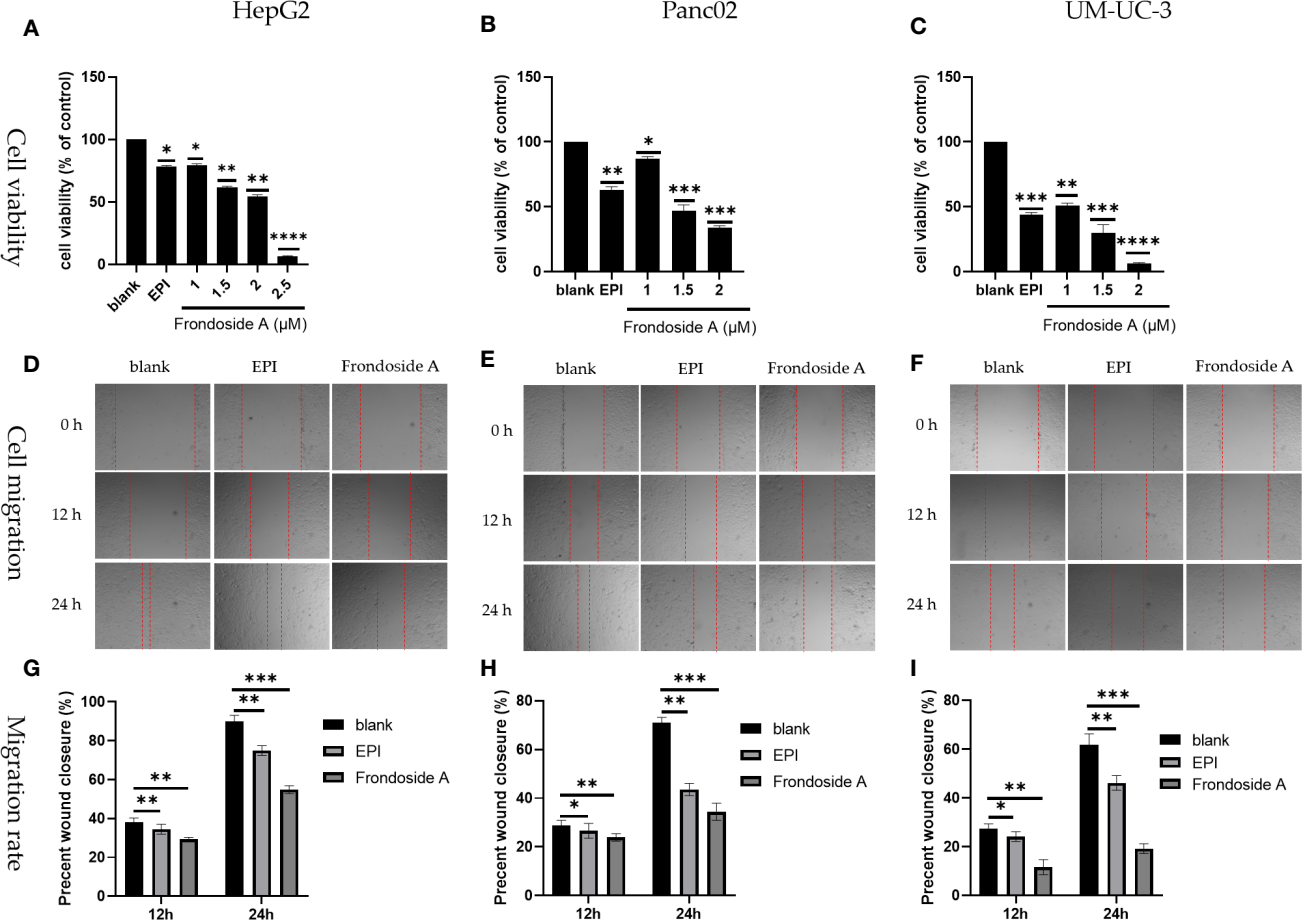
Inhibition of tumor cell proliferation and migration by Frondoside A. **(A–C)** Logarithmic growth HepG2, Panc02, and UM-UC-3 cells were treated with EPI and Frondoside A for 48 h. **(D–F)** Figures of cell migration of HepG2, Panc02, and UM-UC-3 respectively were confluent mono-layers cultured in the presence or absence (control) with the IC50 concentrations of Frondoside A for 0, 12, and 24 h, and EPI (10 μM) as a positive group. **(G–I)** The migration rate of HepG2, Panc02, and UM-UC-3, respectively. Determination of cells as described in Materials and methods. All experiments were repeated at least three times. Data for SD ± means, *p < 0.05, * *p < 0.01, * * * p < 0.001, * * * *p < 0.0001.

As shown in **Figures 2A to C**, Frondoside A exhibited a dose-dependent reduction in the cell viability in HepG2, Panc02, and UM-UC-3 cell lines. The IC_50_ values of Frondoside A for HepG2, Panc02, and UM-UC-3 were 1.5 μM, 1.5 μM, and 1 μM, respectively.

As shown in **Figures 2D to E**, both Frondoside A and EPI treatments significantly inhibited cell migration and reduced the proliferation of HepG2, Panc02, and UM-UC-3. In addition, Frondoside A exhibited a more conspicuous suppression of cell migration than EPI (**Figures 2G–I**) The migration rate of HepG2, Panc02, and UM-UC-3, respectively.

### 3.2 Identification of DEGs

**Figure 3:**
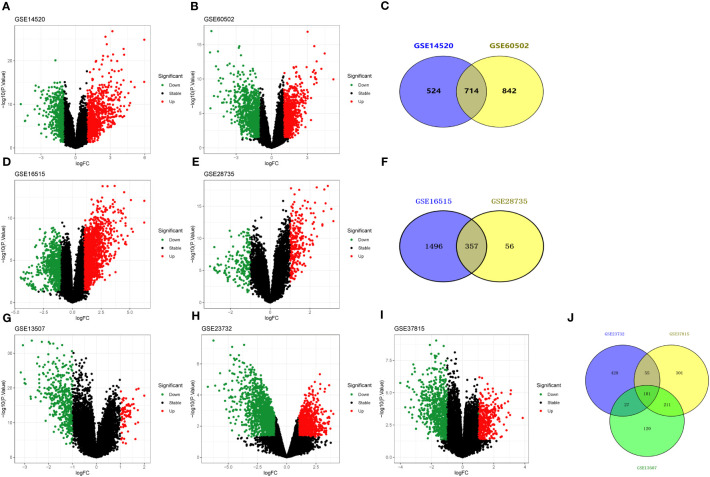
Volcano and Venn diagrams of differentially expressed genes. **(A, B)** Volcano plot showing DEGs in liver cancer tissues and non-tumor samples in GSE14520 and GSE60502 datasets, respectively. **(C)** Venn diagram showing the overlapping DEGs from GSE14520 and GSE60502 datasets. **(D, E)** Volcano plot showing DEGs in pancreatic cancer tissues and non-tumor samples in GSE16515 and GSE28735 datasets, respectively. **(F)** Venn diagram showing the overlapping DEGs from GSE16515 and GSE28735 datasets. **(G–I)** Volcano plot showing DEGs in bladder cancer tissues and non-tumor samples in GSE13507, GSE23732, and GSE37815 datasets, respectively. **(J)** Venn diagram showing the overlapping DEGs from GSE13507, GSE23732, and GSE37815 datasets. Red dots indicate genes highly induced in cancer; green dots indicate genes greatly reduced in cancer; black dots indicate non-DEGs.

GEO is a free database of microarray/gene profiles and next-generation sequencing, from which liver cancer and normal or adjacent liver tissue gene expression profiles of GSE14520 and GSE60502 were obtained. Using *adj. p* < 0.05 and |log_2_FC|> 1 as cut-off criteria, from the microarray data of GSE14520 and GSE60502, 1238 (573 up-regulated and 665 down-regulated) and 1556 (794 up-regulated and 762 down-regulated) DEGs were extracted, respectively (**Figures 3A, B**). After integrated bioinformatical analysis, a total of 714 genes were identified from the two profile datasets (**Figure 3C**).

Subsequently, mRNA expression profiles in human pancreatic tissues and adjacent non-cancerous tissues were analyzed using the GEO databases (i.e., GSE16515 and GSE28735). In GSE16515, 1853 DEGs (i.e., 1364 up-regulated and 489 down-regulated) had significant changes in pancreatic cancer tissues (**Figure 3D**). In GSE28735, 413 DEGs (i.e., 256 up-regulated and 157 down-regulated) had significant changes in pancreatic cancer tissues (**Figure 3E**). Finally, a total of 357 con-DEGs were obtained for further investigation (**Figure 3F**).

Similarly, mRNA expression profiles in human bladder cancer tissues and adjacent non-cancerous tissues were also analyzed using the GEO databases (GSE13507, GSE23732, and GSE37815). For GSE13507, there were 459 DEGs (i.e., 63 up-regulated and 396 down-regulated) with significant changes in bladder cancer tissues (**Figure 3G**). For GSE23732, there were 611 DEGs (i.e., 102 up-regulated and 509 down-regulated) significant changes in bladder cancer tissues (**Figure 3H**). For GSE37815, there were 668 DEGs (i.e., 190 up-regulated and 478 down-regulated) significant changes in bladder urothelial carcinoma tissues (**Figure 3I**). As a result of the analysis above, a total of 101 genes were used for further investigation (**Figure 3J**).

### 3.3 Enrichment analysis

**Figure 4:**
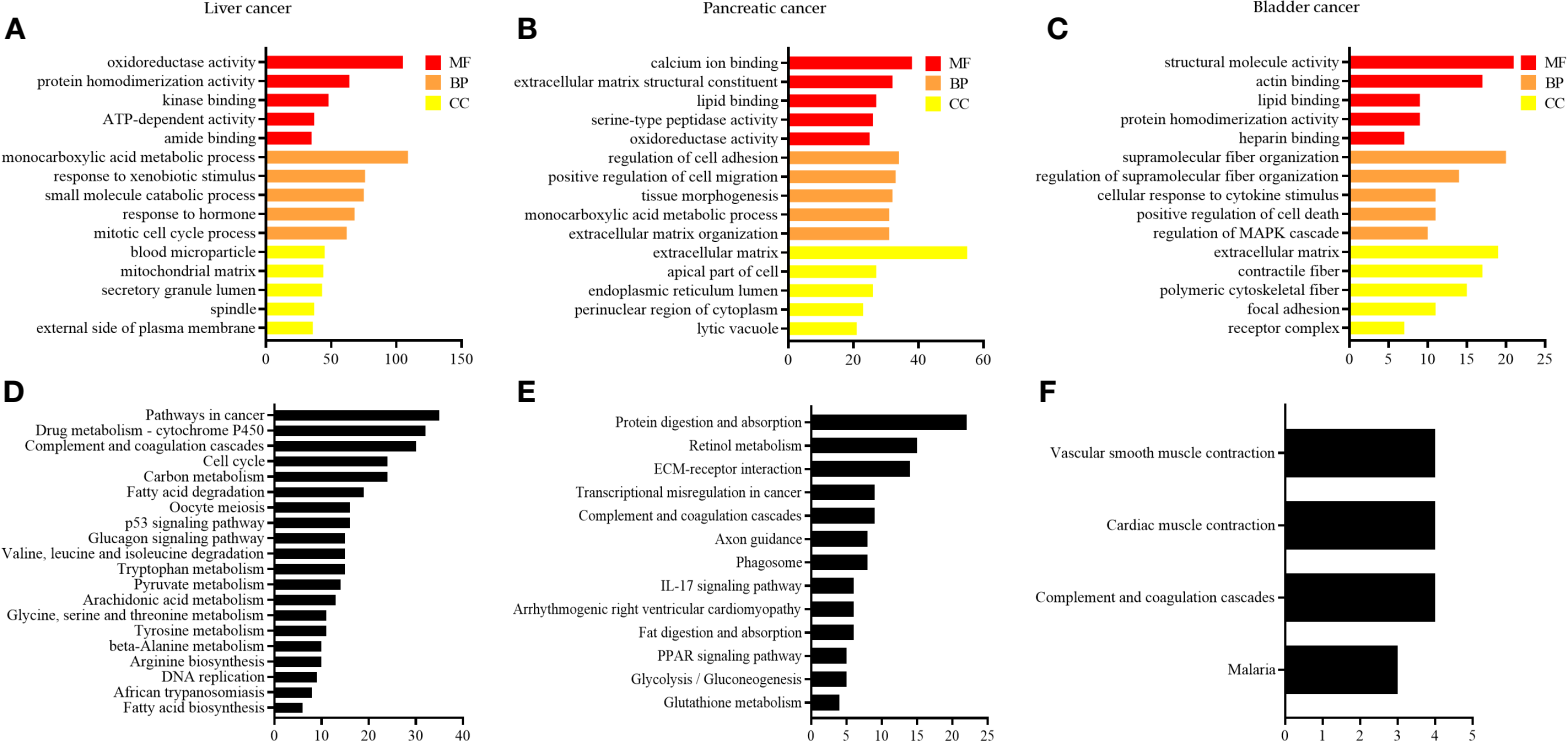
GO and KEGG enrichment analysis of DEGs. **(A–C)** GO enrichment in liver cancer, pancreatic cancer, and bladder cancer, respectively; and **(D–F)** KEGG pathway analysis of liver, pancreatic, and bladder cancers, respectively.

To investigate the biological functions of DEGs, functional annotation and pathway enrichment analysis were conducted using the Metascape database. The results were visualized by generating bar graphs with GraphPad Prism 9.0 software. GO (Gene Ontology) analyses are shown in **Figures 4A–C**, and KEGG analyses are shown in **Figures 4D–F**. The results of GO analysis of DEG in liver cancer are shown in **Figure 4A**. The most important biological processes (BPs) of DEGs were “monocarboxylic acid metabolic process”, “response to xenobiotic stimulus”, “small molecule catabolic process”, “response to the hormone”, and “mitotic cell cycle process”. The molecular functions (MFs) are mainly enriched in “oxidoreductase activity”, “protein homodimerization activity”, “kinase binding”, “ATP-dependent activity”, and “amide binding”. The cellular component (CC) analysis revealed that the differentially expressed genes (DEGs) were predominantly associated with cellular components such as “blood microparticle”, “mitochondrial matrix”, “secretory granule lumen”, “spindle”, and “external side of the plasma membrane”. KEGG pathway analysis shows that the DEGs are significantly involved in several key pathways, including the “pathway in cancer”, “drug metabolism-cytochrome P450 pathway”, “complement and coagulation cascades”, “cell cycle”, and “carbon metabolism” (**Figure 4D**).

The results of GO analysis of DEG in pancreatic cancer are shown in **Figure 4B**, wherein the most important BPs include “regulation of cell adhesion”, “positive regulation of cell migration”, “tissue morphogenesis”, “monocarboxylic acid metabolic process”, and “extracellular matrix organization”. The main enrichment of MFs is observed in “calcium ion binding”, “extracellular matrix structural constituent”, “lipid binding”, “serine-type peptidase activity”, and “oxidoreductase activity”. The CC consists mainly of the “extracellular matrix”, “apical part of the cell”, “endoplasmic reticulum lumen”, “perinuclear region of cytoplasm”, and “lytic vacuole”. KEGG pathway analysis reveals that the DEGs plays a crucial in the “protein digestion and absorption”, “retinol metabolism”, “ECM-receptor interaction”, “transcriptional mis-regulation in cancer”, and “complement and coagulation cascades” (**Figure 4E**).

The results of GO analysis of DEG in bladder cancer are shown in **Figure 4C**, and its most important BPs include “supramolecular fiber organization”, “regulation of supramolecular fiber organization”, “cellular response to cytokine stimulus”, “positive regulation of cell death”, and “regulation of MAPK cascade”. The MFs are mainly enriched in “structural molecule activity”, “actin binding”, “lipid binding”, “protein homodimerization activity”, and “heparin-binding”. The CC consists mainly of the “extracellular matrix”, “contractile fiber”, “polymeric cytoskeletal fiber”, “focal adhesion”, and “receptor complex”. KEGG pathway analysis reveals that the DEGs plays a significant role in the “vascular smooth muscle contraction”, “cardiac muscle contraction”, “complement and coagulation cascades”, and “malaria” (**Figure 4F**).

### 3.4 PPI network analysis

**Figure 5:**
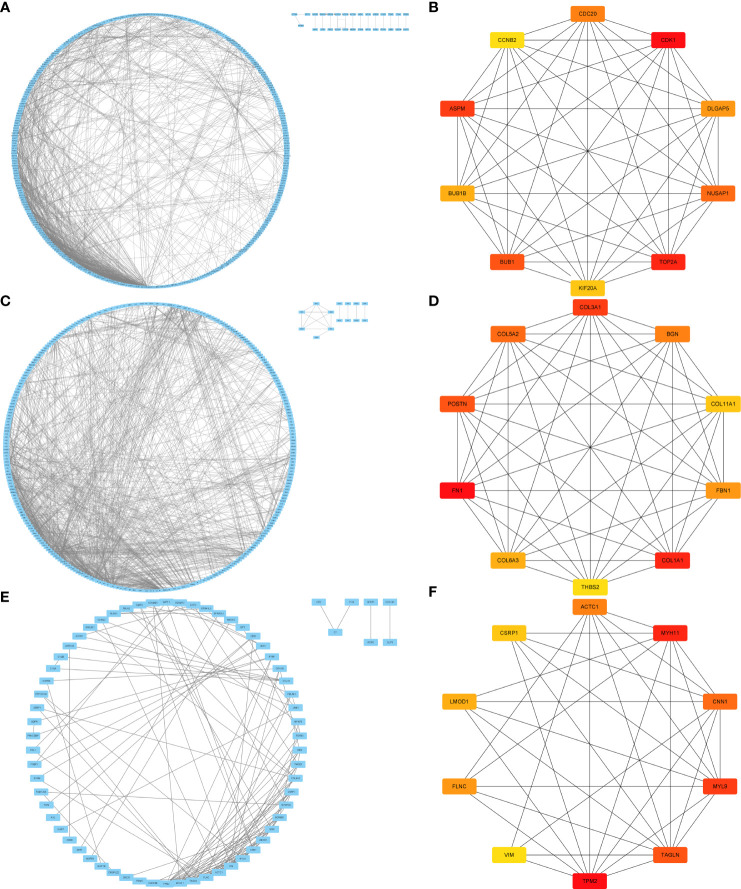
PPI and key target screening of differentially expressed genes. **(A, C, E)** are the PPI figures for liver cancer, pancreatic cancer, and bladder cancer, respectively; **(B, D, F)** are the key targets of liver cancer, pancreatic cancer, and bladder cancer.

using the STRING database, a protein-protein interaction (PPI) network consisting of 714 conserved genes in hepatocellular carcinoma was constructed. The network comprised of 656 nodes and 1461 edges (**Figure 5A**). By considering the degree of connectivity in the PPI network, CytoNCA identified CDK1, TOP2A, ASPM, BUB1, NUSAP1, CDC20, DLGAP5, BUB1B, KIF20A, and CCNB2 as the top ten genes. These genes were tentatively regarded as potential key genes associated with hepatocellular carcinoma, emphasizing the need for further investigation (**Figure 5B**).

Similarly, a PPI network was established based on 357 conserved genes in pancreatic cancer, revealing a network with 86 nodes and 1133 edges (**Figure 5C**). Applying the degree of connectivity analysis, CytoNCA identified ASPM, TOP2A, DLGAP5, TPX2, CENPF, KIF23, MELK, LAMC2, LAMA3, and ANLN as the top ten genes. These genes were provisionally considered as potential key genes associated with pancreatic cancer, warranting further investigation (**Figure 5D**).

Furthermore, a PPI network was constructed based on 101 conserved genes in bladder cancer, consisting of 101 nodes and 131 edges (**Figure 5E**). Based on the degree of connectivity, CytoNCA identified TPM2, MYH11, MYL9, TAGLN, CNN1, ACTC1, FLNC, LMOD1, CSRP1, and VIM as the top ten genes. These genes were preliminarily regarded as potential key genes associated with bladder cancer, necessitating further investigation (**Figure 5F**).

### 3.5 Expression levels of key genes

**Figure 6:**
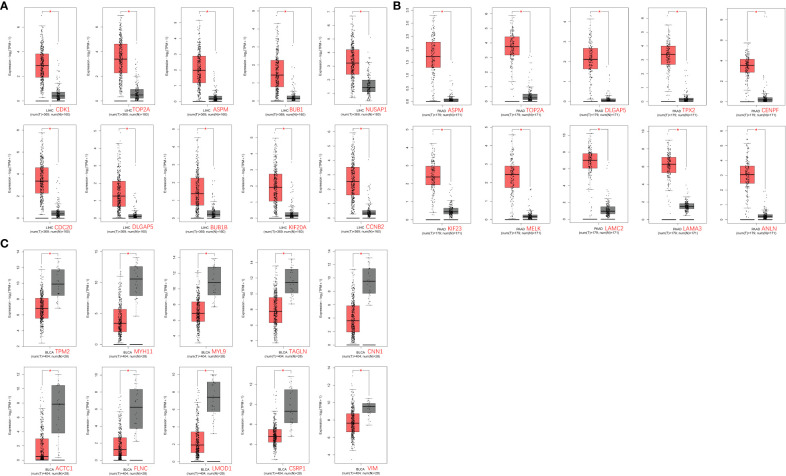
Differential expression analysis of key genes in tumors in the GEPIA2.0 database. **(A)** Liver cancer tissue; **(B)** Pancreatic cancer tissue; and **(C)** Bladder cancer tissue.

The GEPIA2.0 database was used to verify the mRNA expression levels of key genes in tumor and normal tissues, and the results are shown in **Figure 6**. The expression levels of relevant potential key genes were markedly elevated in liver cancer and pancreatic cancer tissues compared to normal tissues (**Figures 6A, B**); conversely, the expression levels of these genes were significantly decreased in bladder cancer tissues compared to normal tissues (**Figure 6C**).

### 3.6 Expression of key genes and their correlation with prognosis

**Figure 7:**
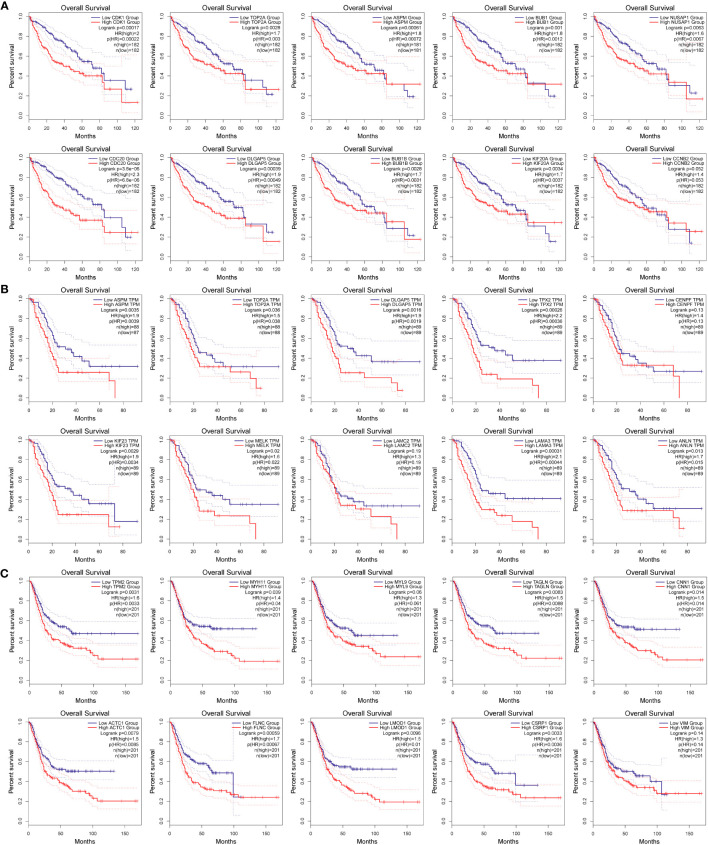
Relationship between target gene expression and survival of tumor patients. **(A–C)** are the total survival time of hepatocellular carcinoma, pancreatic cancer, and bladder cancer, respectively.

To investigate whether the variation in mRNA expression levels of key genes affects the correlation between overall survival (OS) in cancer patients, further analysis was conducted. The GEPIA2.0 online platform was utilized to conduct Kaplan-Meier survival analysis, to explore the potential associations between key genes and overall survival (OS) in tumor patients. The analysis revealed that a majority of the genes exhibited a significant decrease in survival rate (*p* < 0.05, **Figure 7**). High levels of expression of CDK1 (*p* = 0.00022), TOP2A (*p* = 0.003), ASPM (*p*= 0.00072), BUB1 (*p* = 0.0012), NUSAP1 (*p* = 0.0067), CDC20 (*p* = 6.8E-06), DLGAP5 (*p*= 0.00049), BUB1B (*p* = 0.0031), and KIF20A (*p* = 0.0037) were found to be significantly associated with the OS in patients with liver cancer (*p* < 0.05); while CCNB2 (*p* = 0.053) expression was not relevant to survival, as shown in **Figure 7A**.

High levels of expression of ASPM (*p* = 0.0039), TOP2A (*p* = 0.038), DLGAP5 (*p* = 0.0019), TPX2 (*p* = 0.00036), KIF23 (*p* = 0.0034), MELK (*p* = 0.022), LAMA3 (*p* = 0.00044), and ANLN (*p* = 0.015) were significantly correlated with the OS of patients with pancreatic cancer, while CENPF (*p* = 0.13) and LAMC2 (*p* = 0.19) expression were not relevant to survival, as shown in **Figure 7B**.

High levels of expression of TPM2 (*p* = 0.0033), MYH11 (*p* = 0.04), TAGLN (*p* = 0.0088), CNN1 (*p* = 0.014), ACTC1 (*p* = 0.0085), FLNC (*p* = 0.00067), LMOD1 (*p* = 0.01), and CSRP1 (*p* = 0.0036) were found to be significantly associated with OS in patients with bladder cancer, while MYL9 (*p* = 0.061) and VIM (*p* = 0.14), as shown in **Figure 7C**.

### 3.7 Key gene expressions are correlated with immune infiltration and immune cells

The study investigated the association between the expression of key genes and six types of infiltrating immune cells, namely B cells, CD8^+^ T cells, CD4^+^ T cells, macrophages, neutrophils, and dendritic cells, in order to determine their potential as independent predictors of cancer treatment and prognosis.

**Figure 8:**
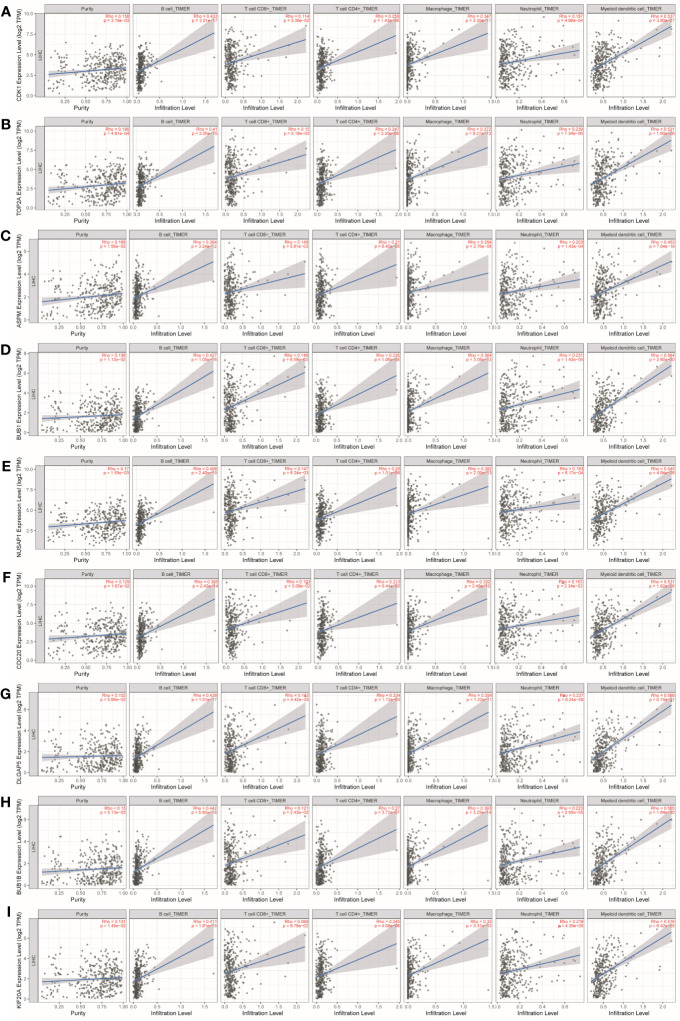
Analysis of key gene expression in various immune cells in liver cancer. The correlation between the expression of **(A)** CDK1, **(B)** TOP2A, **(C)** ASPM, **(D)** BUB1, **(E)** NUSAP1, **(F)** CDC20, **(G)** DLGAP5, **(H)** BUB1B, and **(I)** KIF20A and the degree of immune invasion in liver cancer was investigated using the TIMER database (http://timer.cistrome.org). The Rho value, which indicates Pearson’s correlation coefficient, was used to evaluate the relationship between the genes and immune cells. When |Rho| > 0.1 and *p* < 0.05, it was considered that a correlation existed between the genes and immune cells. In general, the shape of the curve varied depending on the Rho value. When Rho < 0.5, the curve was elliptical; when Rho = 0.5, the curve was parabolic; and when Rho > 0.5, the curve was hyperbolic. These findings provide insights into the potential roles of these key genes in immune invasion in liver cancer.

As shown in **Table 1** and **Figure 8**, CDK1, TOP2A, ASPM, BUB1, NUSAP1, CDC20, DLGAP5, BUB1B, and KIF20A expression were positively correlated with infiltration of B cells, CD4^+^ T cells, macrophages, neutrophils, and dendritic cells. CDK1, TOP2A, ASPM, BUB1, NUSAP1, DLGAP5, and BUB1B expression were positively correlated with infiltration of CD8^+^ T cells.

**Table 1:** Analysis of key gene expression in various immune cells in liver, pancreatic, and bladder cancers.

Cancers	Targets	B cells		CD8^+^ T cells		CD4^+^ T cells		macrophages		neutrophils		dendritic cells	
		Rho	*p*	Rho	*p*	Rho	*p*	Rho	*p*	Rho	*p*	Rho	*p*
Liver cancer	CDK1	0.433	3.51e-17	0.114	3.36e-02	0.256	1.43e-06	0.347	3.30e-11	0.187	4.96e-04	0.537	3.80e-27
	TOP2A	0.41	2.05e-15	0.15	5.18e-03	0.247	3.30e-06	0.372	9.27e-13	0.239	7.34e-06	0.531	1.60e-26
	ASPM	0.364	3.24e-12	0.148	5.81e-03	0.21	8.45e-05	0.294	2.76e-08	0.203	1.43e-04	0.453	7.64e-19
	BUB1	0.427	1.05e-16	0.146	6.58e-03	0.235	1.06e-05	0.364	3.05e-12	0.231	1.43e-05	0.564	2.60e-30
	NUSAP1	0.459	2.40e-19	0.147	6.24e-03	0.26	1.01e-06	0.382	2.09e-13	0.183	6.17e-04	0.545	4.94e-28
	CDC20	0.395	2.42e-14	0.103	5.68e-02	0.213	6.44e-05	0.332	2.48e-10	0.163	2.34e-03	0.531	1.62e-26
	DLGAP5	0.439	1.01e-17	0.153	4.42e-03	0.234	1.12e-05	0.354	1.22e-11	0.237	8.24e-08	0.568	6.74e-31
	BUB1B	0.442	5.82e-18	0.121	2.43e-02	0.27	3.72e-07	0.393	3.25e-14	0.223	2.95e-05	0.565	1.64e-30
	KIF20A	0.411	1.81e-15	0.089	9.76e-02	0.245	4.08e-06	0.33	3.31e-10	0.218	4.39e-05	0.516	6.42e-25
Pancreatic cancer	ASPM	0.28	2.06e-04	0.017	8.25e-01	-0.155	4.23e-02	-0.045	5.60e-01	0.13	8.94e-02	0.12	1.19e-01
	TOP2A	0.276	2.52e-04	0.106	1.68e-01	-0.255	7.47e-04	0.062	4.19e-01	0.186	1.49e-02	0.207	6.52e-03
	DLGAP5	0.282	1.83e-04	0.054	4.81e-01	-0.171	2.51e-02	-0.066	3.94e-01	0.142	6.31e-02	0.177	2.08e-02
	TPX2	0.297	8.06e-05	-0.023	7.62e-01	-0.203	7.71e-03	-0.072	3.52e-01	0.067	3.81e-01	0.089	2.45e-01
	KIF23	0.286	1.53e-04	0.084	2.72e-01	-0.183	1.67e-02	-0.002	9.78e-01	0.176	2.14e-02	0.195	1.05e-02
	MELK	0.285	1.54e-04	-0.033	6.71e-01	-0.092	2.30e-01	-0.07	3.64e-01	0.077	3.19e-01	0.082	2.88e-01
	ANLN	0.243	1.29e-03	0.019	8.01e-01	-0.22	3.86e-03	0.031	6.91e-01	0.125	1.03e-01	0.172	2.41e-02
Bladder cancer	TAGLN	-0.178	5.98e-04	0.039	4.5e-01	0.204	8.25e-05	0.28	4.93e-08	0.005	9.31e-01	0.079	1.29e-01
	CNN1	-0.171	9.79e-04	-0.028	5.96e-01	0.22	2.05e-05	0.255	7.45e-07	-0.025	6.29e-01	-0.022	6.71e-01
	ACTC1	-0.151	3.68e-03	0.026	6.15e-01	0.159	2.16e-03	0.276	7.69e-08	0.014	7.90e-01	0.031	5.49e-01
	FLNC	-0.147	4.79e-03	0.107	4.00e-01	0.167	1.33e-03	0.338	2.78e-11	0.095	7.00e-01	0.095	6.79e-01
	LMOD1	-0.129	1.33e-02	-0.088	9.13e-01	0.252	9.66e-07	0.303	2.84e-09	-0.044	4.01e-01	-0.087	9.47e-01

**Figure 9:**
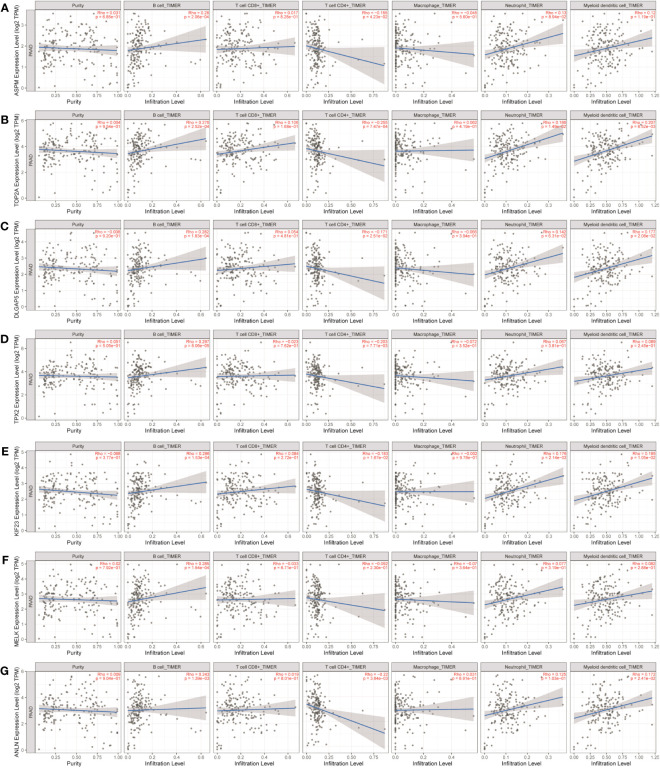
Analysis of key gene expression in various immune cells in pancreatic cancer. The relationship between the expression of **(A)** ASPM, **(B)** TOP2A, **(C)** DLGAP5, **(D)** TPX2, **(E)** KIF23, **(F)** MELK, and **(G)** ANLN and the degree of immune invasion in pancreatic cancer was analyzed using the TIMER database (http://timer.cistrome.org). The Rho value, which indicates Pearson’s correlation coefficient, was used to evaluate the relationship between the genes and immune cells. When |Rho| > 0.1 and *p* < 0.05, it was considered that a correlation existed between the genes and immune cells. The shape of the curve varied depending on the Rho value, with smaller Rho values resulting in smoother curves, while larger Rho values resulted in fuller curves. Elliptical curves were observed when Rho < 0.5, parabolic curves when Rho = 0.5, and hyperbolic curves when Rho > 0.5. These findings provide insights into the potential roles of these key genes in immune invasion in pancreatic cancer.

As shown in **Table 1** and **Figure 9**, ASPM, TOP2A, DLGAP5, TPX2, KIF23, MELK, and ANLN were positively correlated with infiltration of B cells. ASPM, TOP2A, DLGAP5, TPX2, KIF23, and ANLN were negatively correlated with infiltration of CD4^+^ T cells. TOP2A and KIF23 were positively correlated with infiltration of neutrophils. TOP2A, DLGAP5, KIF23, and ANLN were positively correlated with infiltration of dendritic cells.

**Figure 10:**
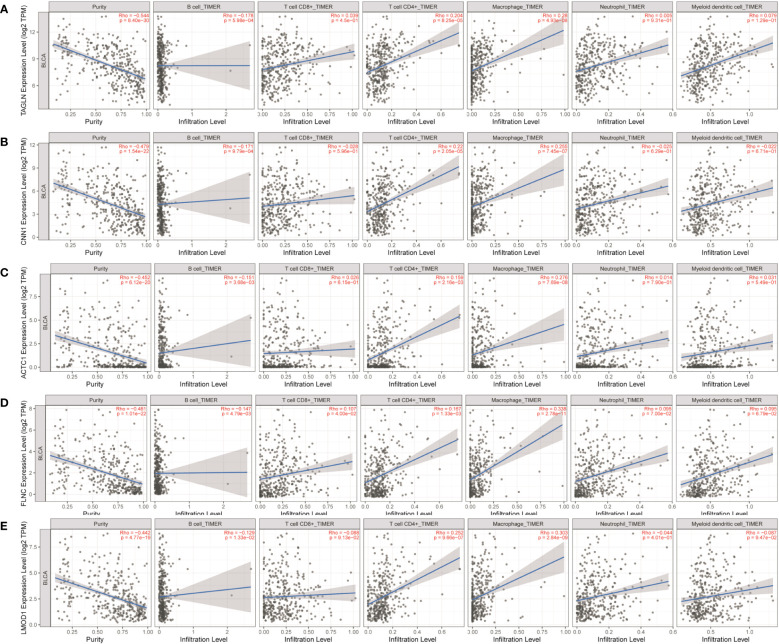
Analysis of key gene expression in various immune cells in bladder cancer. The relationship between the expression of **(A)** TAGLN, **(B)** CNN1, **(C)** ACTC1, **(D)** FLNC, and **(E)** LMOD1 and the degree of immune invasion in bladder cancer was analyzed using the TIMER database (http://timer.cistrome.org). The Rho value, which indicates Pearson’s correlation coefficient, was used to evaluate the relationship between the genes and immune cells. When |Rho| > 0.1 and *p* < 0.05, it was considered that a correlation existed between the genes and immune cells. The shape of the curve varied depending on the Rho value, with smaller Rho values resulting in smoother curves, while larger Rho values resulted in fuller curves. Elliptical curves were observed when Rho < 0.5, parabolic curves when Rho = 0.5, and hyperbolic curves when Rho > 0.5. These findings provide insights into the potential roles of these key genes in immune invasion in bladder cancer.

As shown in **Table 1** and **Figure 10**, TAGLN, CNN1, ACTC1, FLNC, and LMOD1 were negatively correlated with infiltration of B cells. TAGLN, CNN1, ACTC1, FLNC, and LMOD1 were positively correlated with infiltration of CD4^+^ T cells, and macrophages.

### 3.8 Molecular docking

**Figure 11:**
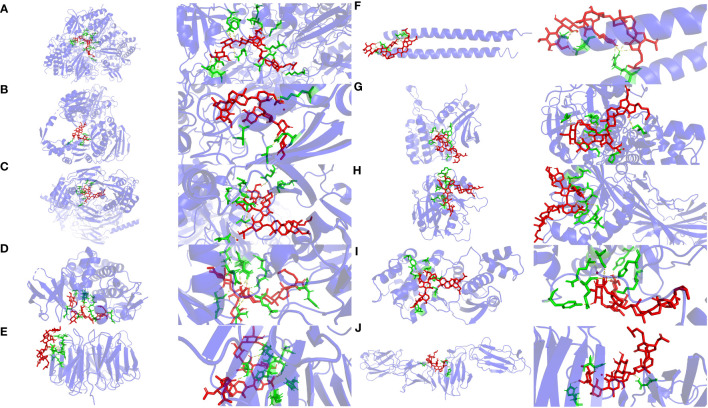
Molecular docking diagrams of Frondoside A and key genes. The yellow, red, green, and light blue represent the hydrogen bond, Frondoside A, amino acid residues, and target protein, respectively. **(A)** CDK1, **(B)** TOP2A, **(C)** ASPM, **(D)** BUB1, **(E)** CDC20, **(F)** KIF20A, **(G)** TPX2, **(H)** KIF23, **(I)** MELK, and **(J)** FLNC.

Molecular docking can be used to explore the optimal binding mode between compounds and targets. Therefore, to further investigate interactions between Frondoside A with its target molecules, CDK1, ASPM, TOP2A, BUB1, CDC20, KIF20A, TPX2, KIF23, MELK, and FLNC molecules were used as molecular docking targets, and the molecular binding free energies listed in **Table 2**. Our results show that the interaction between Frondoside A saponins and key targets via hydrogen-bonding indicated high stability and the binding free energy of the Frondoside A and the ten key proteins were all < -5 kcal/mol. Of these, the affinity by CDK1 performed the best (-10.7 kcal/mol). The 2D docking diagrams of Frondoside A with the receptor targets were visualized using PyMOL software as shown in **Figure 11**.

**Table 2:** Binding Free energy of Frondoside A and targets.

Targets	Frondoside A (kcal/mol)
CDK1	-10.7
ASPM	-10.0
BUB1	-9.2
TOP2A	-9.1
MELK	-9.1
KIF23	-7.7
TPX2	-7.6
FLNC	-6.8
CDC20	-6.3
KIF20A	-5.4

As shown in **Figure 11A**, Frondoside A and CDK1 amino acid residues [LYS-34 (2.3 Å),ARG-36 (3.1 Å and 3.4 Å),GLU-163 (2.6 Å),GLU-209 (3.4 Å),GLN-235 (3.0 Å),ARG-170 (3.1 Å),VAL-174 (2.3 Å),GLU-38(2.9 Å),TYR-181 (3.3 Å),ARG-127 (3.2 Å),THR-47 (3.0 Å) and SER-46 (2.3 Å,3.1 Å and 3.2 Å)] formed fifteen hydrogen bonds at different distances, and Frondoside A-CDK1 had the shortest hydrogen bond with VAL-174 (2.3 Å) and SER-46 (2.3 Å,3.1 Å and 3.2 Å).

As shown in **Figure 11B**, Frondoside A formed six hydrogen bonds at different distances with amino acid residues of TOP2A [ASN-380 (3.4 Å), SER-320 (2.6 Å), GLN-310 (2.7 Å and 3.0 Å) and LYS-357(3.0 Å and 3.3 Å)] and the hydrogen bond between Frondoside A-TOP2A and SER-320 (2.6A) was the shortest.

As shown in **Figure 11C**, Frondoside A formed ten hydrogen bonds at different distances with amino acid residues of ASPM [GLU-68 (2.6 Å),THR-72 (3.1 Å and 3.3 Å),ASN-105 (3.1Å),HIS-109 (3.2 Å),GLU-108 (2.3 Å),GLN-609 (2.8 Å),GLU-621 (2.8Å),SER-600 (3.0Å) and HIS-598 (3.4 Å)] and the hydrogen bond between Frondoside A-ASPM and GLU-108 (2.3 Å) was the shortest.

As shown in **Figure 11D**, Frondoside A formed twelve hydrogen bonds at different distances with amino acid residues of BUB1 [GLN-972 (2.7 Å),ALA-797 (2.4 Å and 2.6 Å),ASP-921(2.0 Å and 3.4 Å), LYS-919 (1.9 Å),SEP-969 (3.1 Å),ASN-922 (2.3 Å),ASP-946 (2.2 Å),GLU-795 (2.2 Å and 2.2 Å) and THR-873 (2.2 Å)] and the hydrogen bond between Frondoside A- BUB1 and LYS-919 (1.9 Å) was the shortest.

As shown in **Figure 11E**, Frondoside A formed ten hydrogen bonds at different distances with amino acid residues of CDC20 [ASP-397 (2.0 Å), ARG-383 (1.9Å), HIS-381 (2.9 Å), ASP-397 (2.2 Å,2.6 Å and 3.2Å), ARG-380 (2.4 Å,2.5 Å and 2.6 Å), SER-378 (2.0 Å)] and the hydrogen bond between Frondoside A- CDC20 and ARG-383 (1.9Å) was the shortest.

As shown in **Figure 11F**, Frondoside A formed ten hydrogen bonds at different distances with amino acid residues of KIF20A [ARG-609 (2.1 Å,2.2 Å,2.5 Å,2.7 Å and 2.8 Å), GLU-603 (2.0 Å) and GLY-600 (3.0Å,3.1 Å,3.1 Å and 3.6 Å)] and the hydrogen bond between Frondoside A- KIF20A and GLU-603 (2.0 Å) was the shortest.

As shown in **Figure 11G**, Frondoside A formed ten hydrogen bonds at different distances with amino acid residues of TPX2[SER-266 (3.0 Å), PRO-191 (2.3Å), TYR-197 (2.2 Å and 3.2Å), ARG-189 (3.0 Å and 3.2Å), ARG-195 (2.8 Å,2.9 Å and 3.4 Å) and SER-20 (3.1 Å)] and the hydrogen bond between Frondoside A- TPX2 and TYR-197 (2.2 Å and 3.2Å) was the shortest.

As shown in **Figure 11H**, Frondoside A formed nine hydrogen bonds at different distances with amino acid residues of KIF23 [ASN-172 (3.3 Å and 3.3Å), TRP-168 (3.0Å), ASN-148 (3.0Å), TRP-149 (2.4Å), ARG-145 (2.6 Å,3.3 Å and 3.1 Å) and ILE-144 (3.3 Å)] and the hydrogen bond between Frondoside A- KIF23 and TYR-197 TRP-149 (2.4Å) was the shortest.

As shown in **Figure 11I**, Frondoside A formed nine hydrogen bonds at different distances with amino acid residues of MELK [TYR-97 (3.2 Å), LYS-145 (3.1Å), HIS-68 (2.9 Å and 3.2Å), SER-118 (3.3Å), ARG-114 (2.4 Å and 3.5 Å), TYR-269 (2.2 Å) and GLU-272 (3.5 Å)] and the hydrogen bond between Frondoside A- MELK and TYR-269 (2.2 Å) was the shortest.

As shown in **Figure 11J**, Frondoside A formed three hydrogen bonds at different distances with amino acid residues of FLNC [ASN-1727 (2.9 Å), ASN-1727 (2.7Å) and HIS-1731 (2.98Å)] and the hydrogen bond between Frondoside A- FLNC and ASN-1727 (2.7Å) was the shortest.

## 4 Discussion

Conventional chemotherapeutic drugs currently in use have given rise to drug resistance due to diverse mechanisms of action. Therefore, new and effective therapeutic agents are urgently needed to treat cancer (14). The targeting of cancer growth, survival, migration, and metastasis pathways using drugs with minimal or no toxicity to normal cells is of significant importance (32). Frontside A has been shown to have anticancer activities in many cancer models, such as bladder cancer, lung cancer, breast cancer (33–36). In the present study, Frondoside A has been demonstrated to exhibit concentration-dependent inhibitory effects on the viability of cancer cells. Frondoside A has been observed to significantly reduce cell migration of HepG2, Panc02, and UM-UC-3 cells in a time-dependent manner at IC_50_ concentrations. Moreover, the inhibitory effects of Frondoside A on cell migration were found to be superior to those of EPI. It is important to note that the development of anti-tumor therapeutic agents that induce apoptosis and inhibit tumor invasion or metastasis are highly desirable (37). The effects of Frondoside A on cell viability or proliferation have been tested using multiple different methods in many different cancers (14, 38, 39). Frondoside A has demonstrated potent growth inhibitory effects on human pancreatic cancer cells, exhibiting an EC50 of approximately 1 µM (38). The observed inhibition of proliferation is accompanied by a significant increase in apoptosis (14, 38). Frondoside A exhibits significant cytotoxicity against urothelial carcinoma cells, with IC_50_ values ranging from 0.55 to 2.33 μM (33, 40). Consistent with our findings, Frondoside A, a marine-derived compound, has demonstrated potential as a therapeutic agent for the treatment of liver, pancreatic, and bladder cancers. While the precise mechanism underlying the anti-cancer effects of Frondoside A remains unclear (14), and the effect of Frondoside A on cell cycle and apoptosis of HepG2, Panc02, and UM-UC-3 require further investigation.

Recently, a study has reported that metformin can induce G2/M arrest and significantly inhibit the proliferation of HCC cells (41). Additionally, metformin has been found to effectively downregulate the expression of CDK1 (41). Several studies have reported significant upregulation of TOP2A mRNA and protein expression in hepatocellular carcinoma (HCC), suggesting that TOP2A is overexpressed in this cancer type and may serve as a potential biomarker for HCC (42). ASPM overexpression has been recognized as a molecular marker that correlates with heightened invasive and metastatic potential in hepatocellular carcinoma (HCC) (43, 44). In contrast, upregulation of BUB1 significantly promoted cell proliferation, while downregulation of BUB1 expression inhibited the proliferation of liver cancer cell lines (45–47). Nucleolar and spindle-associated protein 1 (NUSAP1) is a member of the NUSP1 family of nucleolar-spindle-associated proteins and is involved in spindle microtubule organization (48). Li (49, 50) et al. studied the impact of CDC20 on the progression of hepatocellular carcinoma (HCC) and found that CDC20 expression was elevated in HCC samples. Transfection with CDC20 small interfering RNA in HCC cells led to reduced cellular proliferation and increased cell numbers in the G2/M phase. DLGAP5 (also known as HURP or KI-AA0008) is a cell-cycle-regulated protein that plays a role in tumor development (51). BUB1B, a crucial mitotic spindle checkpoint, is overexpressed in adrenocortical carcinomas (52) and promotes tumor proliferation while inducing radio resistance in glioblastoma (53, 54). KIF20A accumulates in the nucleus during the G2 phase of the cell cycle and promotes both normal and pathological hepatocyte proliferation (55).

ASPM-iII has been shown to selectively regulate cyclin E expression levels and cell cycle progression in PDAC cells (56). TOP2A expression is upregulated in pancreatic cancer tissues compared to non-tumor tissues, and its upregulation is significantly associated with tumor metastasis and shorter survival in pancreatic cancer patients (57–59). Knockdown of TOP2A in pancreatic cancer cell lines inhibits cell proliferation and migration. DLGAP5 expression is significantly elevated in pancreatic cancer tissues and is correlated with patients’ survival and progression-free survival (60). Knockdown of DLGAP5 inhibits pancreatic cancer cell proliferation, invasion, and migration (61). TPX2 has been identified as a prognostic biomarker of KARS-mutant PDAC (62). Knockdown of KIF23 can inhibit pancreatic cell proliferation (63). Silencing of MELK significantly reduces pancreatic cancer development (64), as MELK promotes CDK1 involvement in the cell cycle and cell progression in cancers (65). ANLN expression is significantly upregulated in pancreatic cancer tissues and cell lines and is associated with tumor size, differentiation, TNM stage, lymph node metastasis, distant metastasis, and poor prognosis in pancreatic cancer. ANLN knockdown inhibits several cell-cell adhesions-related genes, including the gene encoding LIM and SH3 protein 1 (LASP1). LASP1 upregulation partially reverses the tumor-suppressive effect of ANLN downregulation on pancreatic cancer cell progression. ANLN contributes to pancreatic cancer progression by regulating the EZH2/miR-218-5p/LASP1 signaling axis (66, 67).

Consistent with our study, previous studies have reported these key genes (TAGLN, CNN1, ACTC1, LMOD1) play important roles in bladder cancer. TAGLN is a recognized actin-binding protein that modulates the dynamics of the actin cytoskeleton (68–70). CNN1 was significantly lowly expressed in BC tissues and cells (70). ACTC1 is responsible for encoding cardiac actin, and a c.G301A mutation in this gene has been shown to be associated with hypertrophic cardiomyopathy, which, in some instances, leads to sudden cardiac death (71). Previous studies showed that ACTC1 is related to cancer prognosis (72, 73), ACTC1 expression is significantly upregulated in glioblastoma and inhibits migration of cancer cells (74), and ACTC1 correlates with the prognosis of glioblastoma and can be used as a novel prognostic marker in glioma (72). LMOD1 belongs to the LMOD family of proteins, which exhibit a striking similarity to actin-capping proteins referred to as Tropomodulins (TMODs) (75). LMOD1, also known as Leiomodin 1, displays widespread expression in a majority of tissues, with particularly elevated levels in the thyroid, skeletal muscle, eye muscle, and ovary (76). Dysregulated expression of LMOD1 may potentially be linked to various pathological conditions (77). LMOD1 was found to be a new gastric cancer biomarker and therapeutic target that induces EMT by regulating the FAK-Akt/mTOR pathway (78).

Molecular docking was used in this study to investigate the mechanism and provide valuable guidance for drug screening and design in future experiments. Based on our molecular investigations, the free energies of Frondoside A and the critical proteins were observed to be below -5 kcal/mol, indicating favorable binding of the aforementioned active ingredients to their respective targets. Studies have shown that Frondoside A can down-regulate CDC20 gene expression, which inhibits the proliferation and migration of cancer cells and promotes cycle arrest and cell apoptosis (79, 80). In addition, Zhang and colleagues have shown that TOP2A deletion can significantly inhibit the proliferation and migration of cancer cells and induce cell apoptosis (81–83). Furthermore, down-regulated expression of CDK1 not only inhibits proliferation and migration of hepatocellular carcinoma cells, but also induces cycle arrest and specific apoptosis of hepatocellular carcinoma cells (84, 85). Finally, overexpression of CNN1 has been shown to inhibit the proliferation, invasion and metastasis of bladder cancer cells (86). Therefore, it is likely that that Frondoside A inhibits the proliferation and metastasis of cancer cells by down-regulating CDK1, CDC20, TOP2A and up-regulating CNN1.It should be noted that the free binding energy between Frondoside A and CDK1 was the lowest, measuring -10.7 kcal/mol. This indicates that Frondoside A may inhibit the development of liver cancer through its effect on the cell cycle (39). The specific mechanisms need to be further investigated.

In addition, Frondoside A may affect immune infiltration by modulating certain targets. These targets may involve activation, recruitment, or inhibition of immune cells. For example, Frondoside A may inhibit the expression of certain pro-inflammatory cytokines in order to reduce the inflammatory response and immune cell infiltration. Alternatively, it may act directly on immune cells, such as T cells or macrophages, affecting their activation and function. This effect may help to improve the tumor microenvironment and enhance anti-tumor effects. However, further studies are needed to validate this hypothesis and determine the specific molecular mechanisms.

## 5 Conclusions

The integration of bioinformatics and molecular docking analyses has enhanced our comprehension of the underlying molecular mechanisms through which Frondoside A exerts its effects in hepatocellular carcinoma, pancreatic cancer, and bladder cancer. Our findings suggest that Frondoside A holds great promise as a potential therapeutic candidate for the treatment of these cancers due to its multi-targeted and multi-mechanistic approach.

## Data availability statement

The original contributions presented in the study are included in the article/**Supplementary Material**. Further inquiries can be directed to the corresponding author.

## Author contributions

GL: Data curation, Formal analysis, Investigation, Methodology, Resources, Software, Validation, Visualization, Writing – original draft. SZ: Data curation, Software, Validation, Visualization, Writing – review & editing. RL: Data curation, Software, Validation, Visualization, Writing – review & editing. XC: Writing – review & editing. LY: Conceptualization, Funding acquisition, Project administration, Supervision, Writing – review & editing.
